# Cloning, Sequencing, and the Expression of the Elusive Sarcomeric TPM4*α* Isoform in Humans

**DOI:** 10.1155/2016/3105478

**Published:** 2016-09-15

**Authors:** Dipak K. Dube, Syamalima Dube, Lynn Abbott, Ruham Alshiekh-Nasany, Charles Mitschow, Bernard J. Poiesz

**Affiliations:** Department of Medicine, SUNY Upstate Medical University, 750 East Adams Street, Syracuse, NY 13210, USA

## Abstract

In mammals, tropomyosin is encoded by four known TPM genes (TPM1, TPM2, TPM3, and TPM4) each of which can generate a number of TPM isoforms via alternative splicing and/or using alternate promoters. In humans, the sarcomeric isoform(s) of each of the TPM genes, except for the TPM4, have been known for a long time. Recently, on the basis of computational analyses of the human genome sequence, the predicted sequence of TPM4*α* has been posted in GenBank. We designed primer-pairs for RT-PCR and showed the expression of the transcripts of TPM4*α* and a novel isoform TPM4*δ* in human heart and skeletal muscle. qRT-PCR shows that the relative expression of TPM4*α* and TPM4*δ* is higher in human cardiac muscle. Western blot analyses using CH1 monoclonal antibodies show the absence of the expression of TPM4*δ* protein (~28 kDa) in human heart muscle. 2D western blot analyses with the same antibody show the expression of at least nine distinct tropomyosin molecules with a mass ~32 kD and above in adult heart. By Mass spectrometry, we determined the amino acid sequences of the extracted proteins from these spots. Spot “G” reveals the putative expression of TPM4*α* along with TPM1*α* protein in human adult heart.

## 1. Introduction

**Figure 1:**
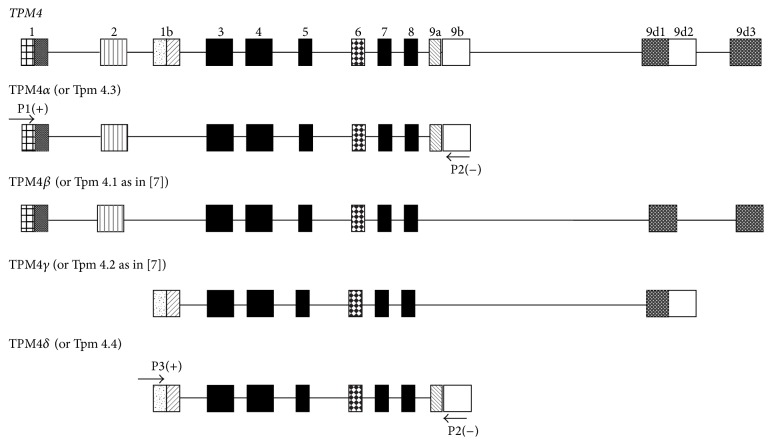
Alternative splicing pattern of the* TPM4* gene in humans. Exon composition of the* TPM4* gene is derived from the published documents (top) and the recently submitted data in GenBank. Although the splice variants TPM4*β* (NM_001145160) and TPM4*γ* (NM-003290.2) are known for some time, the cartoons of the splice variants of TPM4*α* and TPM4*δ* are generated with the information of the recently submitted sequences of the corresponding predicted isoforms in GenBank as well as the results from the present study. Exon locations of primers P1(+), P2(−), and P3(+) are shown.

Tropomyosin (TPM) is a coiled-coil dimer protein that plays important role(s) in regulating muscle contraction in conjunction with other sarcomeric proteins like actin, troponins, tropomodulin, and so forth. TPM also provides structural stability to actin filaments, thereby modulating cytoskeleton function. In vertebrates, there are four TPM genes, designated as* TPM1*,* TPM2*,* TPM3*, and* TPM4*, except for zebrafish where six* TPM* genes are present [1–10]. Each of the four genes generates a large number of TPM isoforms via alternative splicing and/or using different promoters. In mammals, the exon and intron organization of* TPM* genes are very similar, if not identical [6]. The rodent TPM4 gene has lost the use of exons 1a and 2; the high molecular weight isoforms encoding 284 amino acid residues such as TPM4*α* and TPM4*β* are not expressed as they are in nonmammalian species. It was speculated for a long time that the* TPM4* gene in humans as in rodents does not encode sarcomeric isoform with exon 9a/b [2, 3]. The first report on the expression of a high molecular weight TPM4 protein was reported in human ovary tumor tissues in 2004 (accession number AK023385). This isoform, defined as TPM4*β*, contains exons 1a, 2, 3, 4, 5, 6, 7, 8, and 9d (Figure 1), which differs from the sarcomeric TPM4*α* isoform containing exon 9a/b instead of 9d. It is to be noted that exon 9a but not 9d encodes the peptide that is essential for binding TPM with troponins [2, 3], essential components of thin filament. Recently, two predicted sequences (derived from a genomic sequence) of the two different isoforms of the human* TPM4* gene containing exon 9a have been reported in GenBank (on March 12, 2015). One of the predicted isoforms is TPM4*α* (as in avian and amphibians) containing exons 1a, 2, 3, 4, 5, 6b, 7, 8, and 9a/b encoding 284 amino acids (accession number XM_006722865). The other one may encode a low molecular weight novel protein with 248 amino acids consisting of exons 1b, 3, 4, 5, 6, 7, 8, and 9a/b (accession number XM_005260042.2). In this study, for the first time we confirmed the expression of these two new transcripts, viz TPM4*α* (or Tpm4.3 (8)) and TPM4*δ* (Tpm 4.4 (8)) by RT-PCR with the RNA from human adult heart, fetal heart, and skeletal muscle using two primer-pairs designed from the predicted nucleotide sequences. We confirmed the sequences of both the isoforms by cloning and sequencing of the amplified products. We also determined the relative expression of the two transcripts by qRT-PCR with RNA from human adult heart, fetal heart, and skeletal muscle using isoform specific primer-pairs. For protein expression, we performed 1D and 2D western blot analyses with the extract from adult human heart using the exon 9a-specific monoclonal antibody, CH1. We found nine spots on western blot and the protein was extracted from the spots for mass spectrometry (LC-MS/MS) analyses. The results suggest that spot G may have TPM4*α* along with TPM1*α* protein. To the best of our knowledge, this is the first report of TPM4*α* protein expression in humans.

## 2. Materials and Methods

Human adult and fetal cardiac tissue RNA were obtained from Zyagen (San Diego, CA); adult human heart protein samples were obtained from Imgenex (San Diego, CA).

### 2.1. Conventional RT-PCR

All PCR and post-PCR experiments were done by different personnel in in different buildings using separate equipment and separate ventilation so as to avoid carryover contamination. For RT-PCR, 0.5 *μ*g of RNA in a total volume of 40 *μ*L was used to synthesize cDNA with SuperScript® II (Life Technologies, Grand Island, NY) and oligo-dT primers following the manufacturer's specifications. For each PCR 3 *μ*L of cDNA was used and TPM4*α* and TPM4*δ* RNA were amplified as previously described [11, 12]. The nucleotide sequences of the primer-pairs for amplification of TPM4*α* and TPM4*δ* are given in Table 1. It is to be noted that the negative primer for amplification of TPM4*α* and TPM4*δ* is the same. The positive primers for TPM4*α* and TPM4d are from exon 1a and exon 1b, respectively. The PCR amplified DNAs were run in an agarose gel and the ethidium bromide stained bands were excised from the gel and DNA was extracted from the excised bands using a MinElute Gel extraction kit from Qiagen (Valencia, CA) following the manufacturer's direction. Part of the DNA was used for sequencing and part of the gel extracted DNAs was ligated and cloned into the T/A cloning vector (Life Technologies) following our published protocol [11, 12]. Positive clones were identified by PCR using the above-mentioned specific primer-pairs previously described. Vectors or constructs were grown in* E. coli*, and the DNA was extracted using Qiagen Miniprep kit (Valencia, CA). The isolated DNA was sequenced (Cornell University Life Science Core Laboratories Center, Ithaca, NY). Each clone was sequenced twice in both directions. It is to be noted that the primer-pair we have used for TPM4*α* and TPM4*δ* is very specific for the respective isoform.

### 2.2. Real-Time Quantitative RT-PCR

qRT-PCR analysis of cDNA template was performed using the LightCycler 480 Real-Time PCR System. Reactions were carried out in a 384-well plate using the LightCycler 480 SYBR Green I Master kit (Roche). Briefly, each well contained a total volume of 10 *μ*L reaction solution, of which 2 *μ*L was.

cDNA template and 8 *μ*L were SYBR green mix (5 *μ*L 1x SYBR green master mix, 2.8 *μ*L of PCR-grade water, and 0.2 *μ*L of 10 *μ*M primer-pair). Primers for real-time PCR for TPM4*α* and TPM4*δ* are listed in Table 1.

In this case cDNA was made with an oligonucleotide from exon 9a/b (5′-CACCATGTGAGAAGGACAGA-3′) that allowed us to make a gene specific cDNA corresponding to the mRNA containing exon 9/b as in TPM4*α* and TPM4*δ* and would avoid any subsequent nonspecific PCR amplification. For the amplification of TPM4*α* and TPM4*δ*, we have employed the positive primers from exon 1a and exon 1b, respectively, and the same negative primer located in exon 3. Amplification in the absence of cDNA template was also evaluated to insure a lack of signal due to primer dimerization and extension or carryover. All samples and controls were performed in triplicate. Data were analyzed using delta-delta CT (sample delta CT minus comparator delta CT) methods [13–15]. Efficiencies (E) of PCR amplification were determined using dilution series of plasmids and/or human heart cDNAs generated with gene specific primer-pairs as described by Nan et al. [16]. As a result, the primer-pair we have used from each of the isoforms is very specific and does not amplify other isoforms generated from the* TPM4* or any other TPM gene.

### 2.3. Western Blot

10 *μ*g of protein from each sample (human adult and fetal heart) was used for western blot analyses following our published protocol [17]. Primary antibodies included TM311 (Sigma-Aldrich, St. Louis, MO) and CH1 antisarcomeric tropomyosin (DSHB, University of Iowa, Iowa). Secondary antibody was sheep antimouse immunoglobulin HRP (GE Healthcare Bio-Sciences, Pittsburgh, PA).

### 2.4. 2D Western Blot and Mass Spectrometry

2D (two-dimensional) western blot analyses of the commercial human adult heart extract obtained from Zyagen (San Diego, CA) were performed for us by Kendrick Labs, Inc. (Madison, WI). Two-dimensional electrophoresis was performed according to the carrier ampholine method of isoelectric focusing [18, 19] by Kendrick Labs, Inc. (Madison, WI) as described by Wang et al. [20]. The key to 2D Gel loading and sample preparation is given in Table 2. The Coomassie blue-stained PVDF membrane was desktop scanned, destained in 100% methanol, rinsed briefly in Tween-20 Tris-buffered saline (TTBS), and blocked for two hours in 5% nonfat dry milk (NFDM) in TTBS. The blot was incubated in primary antibody (Anti-Tropomyosin-Clone CH1 (Developmental Studies Hybridoma Bank) diluted 1 : 10,000 in 2% NFDM TTBS) overnight and rinsed 3 × 10 minutes in TTBS. The blot was placed in secondary antibody (antimouse IgG-HRP (GE Healthcare, Cat number NA931V, Lot number 9621358) diluted 1 : 2,000 in 2% NFDM TTBS) for two hours, rinsed as above, treated with ECL, and exposed to X-ray film. For the developed X-ray film when superimposed on the Coomassie stained human adult heart protein gel, 9 spots (A–I) were exhibited (as in Figure 7(a)). Nine gel spots were excised, washed, and trypsinized following the published protocols [21–23]. The resulting peptides were extracted twice with 5% formic acid/50 mM ammonium bicarbonate/50% acetonitrile and once with 100% acetonitrile under moderate shaking. The peptide mixture was then dried in SpeedVac, solubilized in 20 *μ*L of 0.1% formic acid/2% acetonitrile.

### 2.5. LC-MS/MS

The peptides mixture was analyzed by reverse phase liquid chromatography (LC) and MS (LC-MS/MS) using a nanoACQUITY UPLC (Micromass/Waters, Milford, MA) coupled to a Q-TOF Ultima API MS (Micromass/Waters, Milford, MA), according to published procedures [22–24]. Briefly, the peptides were loaded onto a 100 *μ*m × 10 mm nanoACQUITY BEH130 C18 1.7 *μ*m UPLC column (Waters, Milford, MA) and eluted over a 150-minute gradient of 2–80% organic solvent (ACN containing 0.1% FA) at a flow rate of 400 nL/min. The aqueous solvent was 0.1% FA in HPLC water. The column was coupled to a PicoTip Emitter SilicaTip nanoelectrospray needle (New Objective, Woburn, MA). MS data acquisition involved survey MS scans and automatic data dependent analysis (DDA) of the top three ions with the highest intensity ions with the charge of 2+, 3+, or 4+ ions. The MS/MS was triggered when the MS signal intensity exceeded 10 counts/second. In survey MS scans, the three most intense peaks were selected for collision-induced dissociation (CID) and fragmented until the total MS/MS ion counts reached 10,000 or for up to 6 seconds each. The entire procedure used was previously described [22–24]. Calibration was performed for both precursor and product ions using 1 pmol GluFib (Glu1-Fibrinopeptide B) standard peptide with the sequence EGVNDNEEGFFSAR and the monoisotopic doubly charged peak with *m*/*z* of 785.84.

### 2.6. Data Processing and Protein Identification

The raw data were processed using ProteinLynx Global Server (PLGS, version 2.4) software as previously described by Darie et al. [22].

## 3. Results

### 3.1. Detection of RNA Expression by RT-PCR and Cloning and Sequencing of TPM4*α* and TPM4*δ* cDNAs

In order to detect the expression of TPM4*α* and TPM4*δ* in human striated muscles, we used conventional RT-PCR with the RNA from adult human heart and skeletal muscles. The primer-pairs were designed from the predicted sequences as reported in the GenBank (accession number XM_006722865.1 for TPM4*α* and XM_005260042.2 for TPM4*δ*). The nucleotide sequences of the primer-pairs designed are presented in Table 1.

**Figure 2 fig2:**
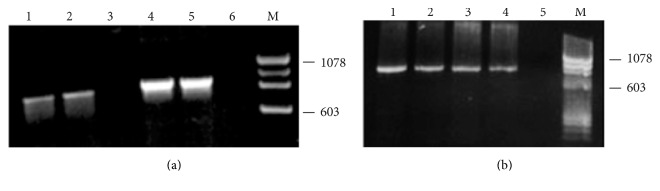
Expression of TPM4 isoforms with exon 9a/b in human heart and skeletal muscle by conventional RT-PCR.* (a) Expression of TPM4α and TPMδ in human fetal and adult hearts as determined*. TPM4*δ* amplified with primer 1(+) and primer 2(−) as shown in Figure 1 and Table 1. The size of the amplified DNA is ~747 bp. Lane 1: adult heart; lane 2: fetal heart; lane 3: primer control. TPM4*α* amplified with primer 1(+) and primer 2(−) as shown in Figure 1 and Table 1. The size of the amplified DNA is ~855 bp. Lane 4: adult heart; lane 5: fetal heart; lane 6: primer control*. (b) Expression of TPM4α in human fetal heart*,* adult heart*,* and adult skeletal muscle*. TPM4*α* amplified with primer 1(+) and primer 2(−) as shown in Figure 1 and Table 1. The size of the amplified DNA is ~855 bp. Lane 1: fetal heart; lane 2: adult heart; lane 3: adult skeletal muscle (source 1); lane 4: adult skeletal muscle (source 2); lane 5: primer control.

Conventional RT-PCR results presented in Figure 2(a) show that TPM4*δ* is expressed in both fetal (lane 1) and adult human heart (lane 2).

Also, the TPM4*α* is expressed in both fetal and adult heart as depicted in lanes 4 and 5, respectively. Again, TPM4a is expressed in fetal heart (lane 1, Figure 2(b)), adult heart (lane 2, Figure 2(b)), and also skeletal muscle (lanes 3 and 4 representing two different sources of adult human skeletal muscle RNA).

**Figure 3:**
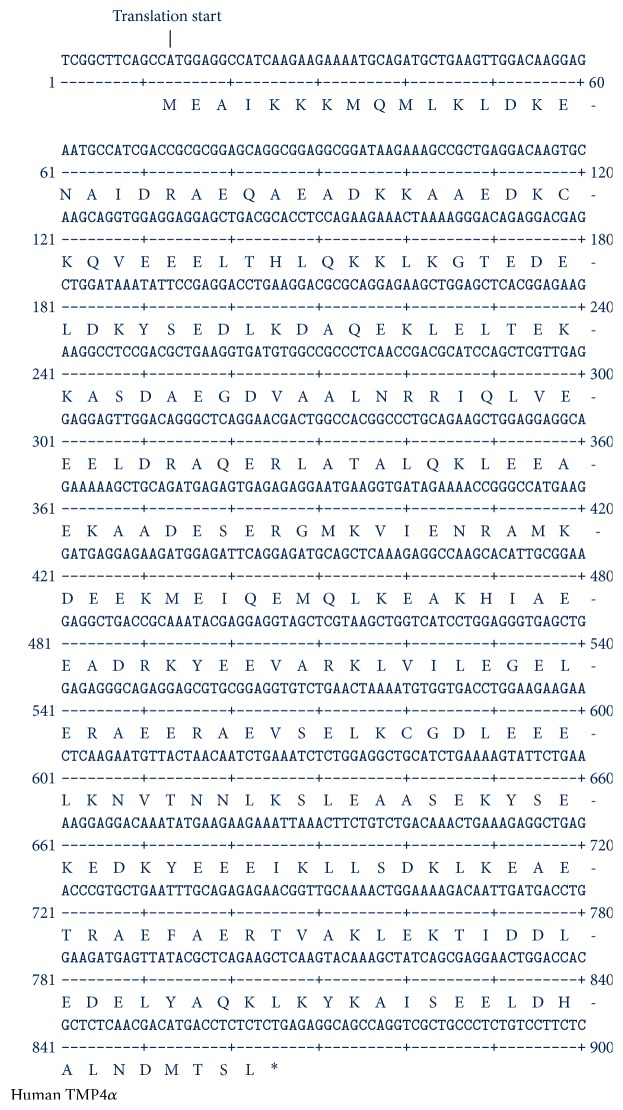
Nucleotide and deduced amino acid sequences of human TPM4*α*. Deduced amino acid sequences are at the bottom of the nucleotide sequences. Symbol of each amino acid residue is placed at the bottom of the first nucleotide of each codon. *∗* signifies Stop codon.

**Figure 4:**
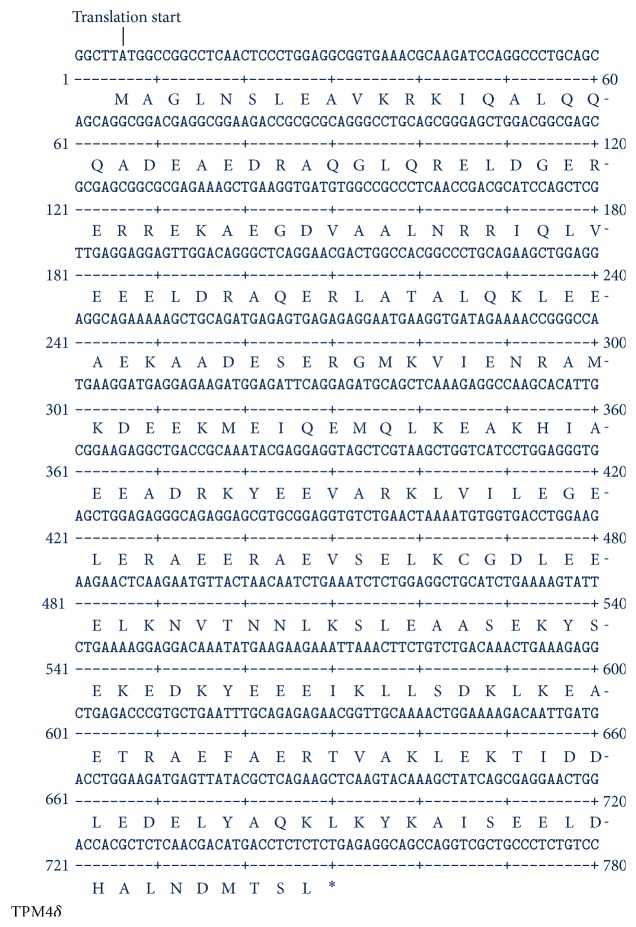
Nucleotide and deduced amino acid sequences of human TPM4*δ*. *∗* signifies Stop codon.

The amplified DNA from the ethidium stained band(s) on agarose gel was extracted and purified using a gel extraction kit from Ambion, Inc. Gel extracted DNA was used subsequently for nucleotide sequence determination as well as cloning into T/A cloning vector (Invitrogen). Direct DNA sequence analyses confirmed the expression of TPM4*α* and TPM4*δ* in both heart and skeletal muscle. DNA sequencing was also performed with the cDNA of each TPM4 isoform cloned in T/A cloning vector. Figures 3 and 4 depict the nucleotide as well as deduced amino acid sequences of TPM4*α* and TPM4*δ*. The sequences are identical with the predicted sequences as submitted in GenBank. When the amino acid sequences of TPM4*α* and TPM1*α* (Acc number NP_001018005) are compared, there are 96.127 percent similarity and 88.028 percent identity between the two sarcomeric isoforms (best fit results not shown). To the best of our knowledge this is the first report on the expression of TPM4*α* and TPM4*δ* in human striated muscles. Comparisons of the amino acid sequences of TPM4*δ* and the known TPM4*γ* indicate that both isoforms encode a 248-amino acid polypeptide with identical sequences except for exon 9. The exon compositions of TPM4*γ* and TPM4*δ* are given in Figure 1. TPM4*γ* contains exons 1b, 3, 4, 5, 6b, 7, 8, and 9d while TPM4*δ* contains exon 9a instead of 9d.

### 3.2. qRT-PCR Analyses to Determine the Relative Expression of TPM4*α* and TPM4*δ* in Human Fetal Heart, Adult Heart, and Adult Skeletal Muscle

**Figure 5:**
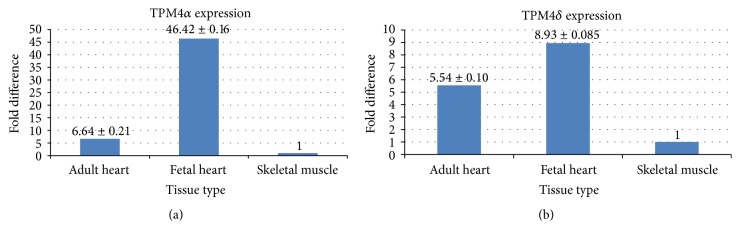
Relative expression of TPM4*α* and TPM4*δ* in human adult and fetal hearts. Fold changes of TPM4*α* and TPM4*δ* in human adult heart, fetal heart, and skeletal muscle. qRT-PCR was carried out in triplicate with isoform specific primer-pairs sequences of which are given in Table 1. (a) TPM4*α* and (b) TPM4*δ*.

The results depicted in Figures 5 and 2(b) confirm that TPM4*α* is expressed in human hearts and skeletal muscle. Interestingly, the expression level of both isoforms is the least in skeletal muscle. However, the relative expression of TPM4*α* and TPM4*δ* is higher in fetal heart compared to adult heart and adult skeletal muscle. The results also indicate that the relative expression of TPM4*α* is much higher compared to TPM4*δ* in all the tissues tested. Please note the difference in scale for TPM4*α* and TPM4*δ* shown in Figure 5.

### 3.3. Western Blot Analyses with the Total Protein Extracts of Human Adult Heart

**Figure 6:**
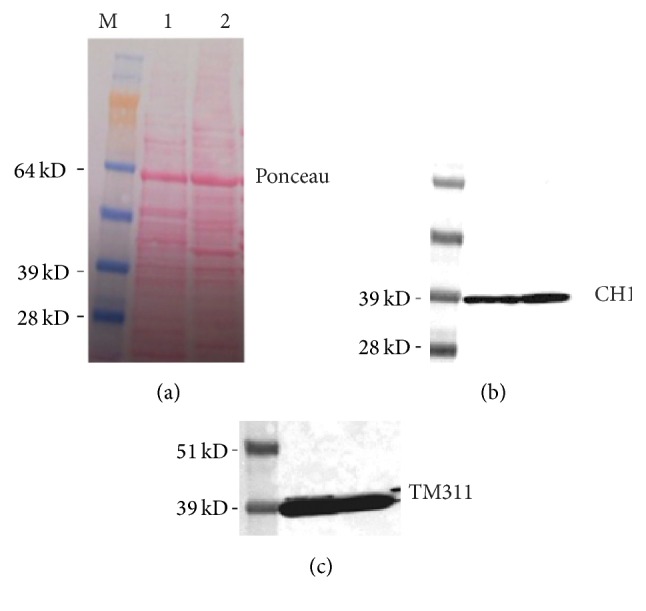
Western blot analysis of protein extracts from human adult and fetal hearts with CH1 and TPM311 antitropomyosin antibodies. (a) Ponceau-stained blot: lane 1: extract of adult heart; lane 2: extract of fetal heart; lane M: molecular weight markers. (b) Blots stained with CH1 monoclonal antibodies that recognize all sarcomeric TPM isoforms as well as TPM4*δ* with exon 9a C-terminus end. (c) Blot stained with TM311 monoclonal antibodies that recognize all high molecular wt TPM isoforms with exon 1a at the N-terminus end. However, it does not recognize TPM4*δ* with exon 1b at the N-terminus end.

In order to explore whether or not TPM4*α* and TPM4*δ* proteins are expressed in human striated muscles, we have employed western blot analyses with the CH1 monoclonal antibody that recognizes an epitope in exon 9a. In other words, CH1 monoclonal antibody recognizes all known sarcomeric TPM isoforms (TPM1*α*, TPM1*κ*, TPM2*α*, TPM3*α*, and TPM4*α*) and the nonsarcomeric TPM4*δ* having a peptide encoded by exon 9a at the C-terminus end. Each of these sarcomeric isoforms contains 284 amino acids and is considered as high molecular wt tropomyosin. Although TPM4*δ* encodes 248 amino acids and has different exon 1, exon 9 is identical to TPM4*α*. Hence, if TPM4*δ* protein is expressed, one should observe a signal at the ~28 kDa region in the western blot. We have employed another monoclonal antitropomyosin antibody TM311, which recognizes all high molecular wt tropomyosin molecules (~32 kDa and above) containing exon 1a. As TPM4*δ*, unlike TPM4*α*, contains exon 1b instead of exon 1a, one should not observe any ~28 kDa signal with TM311 antibodies. The results depicted in Figure 6 (staining with CH1 monoclonal antibodies) show strong signal(s) at ~39 kDa region. The results show the presence of most likely several sarcomeric isoforms, for example, TPM1*α*, TPM1*κ*, TPM2*α*, and TPM3*α*, in human hearts. Without using a TPM4 specific antibody one cannot infer that the signal(s) at the ~39 kDa region indicates the presence of TPM4*α* protein in human cardiac muscles. In addition, we did not detect the presence of low molecular wt tropomyosin like TPM4*δ*. If it was expressed in detectable quantity, we should have picked up a signal. Western blot analyses with TM311 confirm the expression of high molecular wt sarcomeric and nonsarcomeric TPM isoforms in adult and fetal heart tissues. TM311 should not recognize low molecular wt TPM4*δ*, which contains exon 1b rather than exon 1a region [6].

### 3.4. 2D Western Blot Analyses with Protein Extracts of Adult Human Heart with CH1 Monoclonal Antibody That Recognizes All Sarcomeric Tropomyosin Isoforms including TPM4a and the Newly Found TPM4*δ* with Exon 9*α* Polypeptide at the C-Terminus End

**Figure 7:**
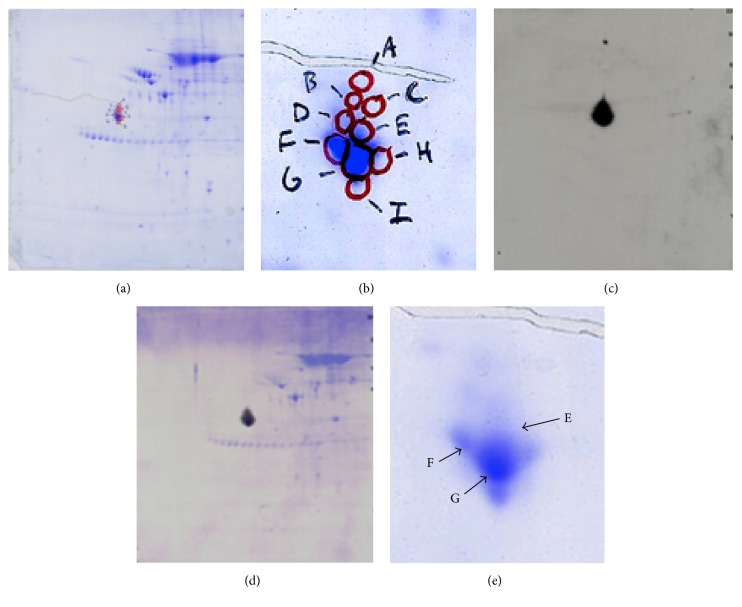
2D gels of commercially available extracts of human adult heart. (a) The Coomassie stained human adult heart protein across the gel. Seven spots A, B, C, D, E, F, and G were identified, which are not visible clearly. (b) This panel represents the enlarged portion of the gel in (a) and shows clearly A–E spots. (c) The PVDF filter was stained with CH1 monoclonal antibody followed by treatment with secondary antibody as mentioned under Materials and Methods and subsequently treated with ECL and exposed to X-ray film. (d) Developed X-ray film was merged on the top of the Coomassie stained blot. (e) Enlarged nine spots (A–I) were marked on the PVDF filter (as in (a) and (b)) and on the duplicate gel. All the spots were marked and excised and were used for extraction of protein for subsequent mass spectrometric analyses. Extracted peptides from spots E, F, and G were analyzed for mass spectrometry as described in the method section.

Two-dimensional western blot analyses were performed with commercially available human heart extract with antisarcomeric antitropomyosin (CH1) antibodies. Figure 7(a) shows the Coomassie blue-stained PVDF membrane before western blotting and Figure 7(b) depicts the signals on the X-ray film exposed on the blot after being treated with ECL (see the method section).  Figure 7(d) represents the developed X-ray film superimposed on the top of the Coomassie blue-stained PVDF membrane (as in Figures 7(a) and 7(b)). Nine spots were identified and marked as A, B, C, D, E, F, G, H, and I (Figures 7(a) and 7(b)). The results indicate that there are at least nine detectable CH1 positive tropomyosin molecules present in the extract from adult human hearts. It is to be noted that each of these spots represents high molecular wt (32 kD and above) tropomyosin protein. However, we have failed to detect the presence of a low molecular wt CH1-positive tropomyosin protein with a molecular mass ~28 kDa like TPM4*δ* in adult human heart.

**Figure 8:**
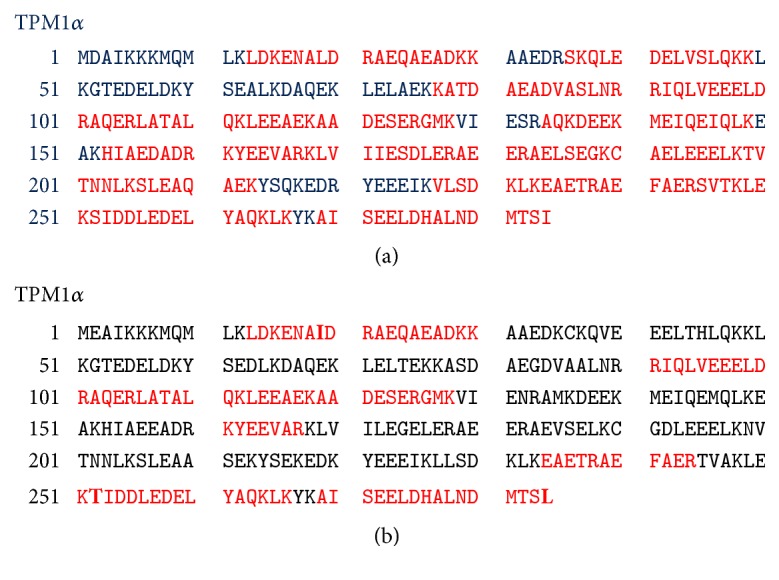
Identification of amino acid sequences from the peptides extracted from spot G after 2D western blot analyses of adult heart extracts with CH1 antibodies. Red color letters indicate the isolated peptides that have been sequenced by mass spectrometry. (a) TPM1*α* and (b) TPM4*α*. Three amino acid residues in exons 1a, 8, and 9a are different in TPM4*α* marked as bold. In case of TPM1*α* 76% (red marked) of the total 284 amino acid residues are being identified. For TPM4*α* it is 38%. It is to be noted that peptide SIDDLEDELYAQKLK in TPM1*α* was identified by two precursor ions with *m*/*z* of 594.07 (3+) and 890.06 (2+) ions. On the other hand, peptide TIDDLEDELYAQKLK in TPM4*α* was identified by one precursor ion with *m*/*z* of 888.06 (2+).

In the next step, each of the protein spots that were separated by 2D PAGE and stained by Coomassie dye was excised and washed and the proteins from the gel were treated/processed according to the published protocol (please see the method section) for LS-MS/MS analyses using nanoACQUITY UPLC (Micromass/Waters, Milford, MA) coupled to a Q-TOF Ultima API MS (Micromass/Waters, Milford, MA). The raw data were processed using ProteinLynx Global Server (PLGS, version 2.4) software. Mascot and PLGS database search provided a list of proteins for each gel band. To eliminate false positive results, for the proteins identified by either one peptide or a mascot score lower than 25, we verified the MS/MS spectra that led to identification of a protein. In spot G, a total of 53 peptide sequences were identified. As shown in Figure 8, the identified peptides cover 76% of TPM1*α* (accession number gi/63252898) and 38% of TPM4*α* protein (accession number GI: 578833543; PREDICTED: tropomyosin alpha-4 chain isoform X1;* Homo sapiens*). It is to be noted that there are not very many differences between TPM1*α* and TPM4*α*. In this study, we found three peptides, containing a total of three amino acid residues, that are specific for TPM4*α* and eight peptides containing eighteen amino acids that are specific for TPM1*α*. The remainders of the identified peptides are identical in both isoforms. For TPM1*α*, peptide in exons 1a, 2, 3, 4, 5, 6, 7, 8, and 9a was identified. For TPM4*α*, peptides in exons 1a, 8, and 9a were identified. Some amino acid changes found in the striated muscle TPM4*α* are also found in skeletal muscle TPM2*α* (gi I 42476296 isoform, which is also expressed in heart). One of such changes is L19I. Again, ^252^T is also present in TPM2*α*. However, the peptide of the TPM4*α* that we have sequenced is quite different from TPM2a peptide. This is true for ^284^L. The peptide containing ^284^L residue from TPM4*α* that we have sequenced (as marked with red color) is different from human TPM2*α* (gi I42476296). The results indicate the potential expression of TPM4*α* protein in human cardiac tissues.

## 4. Discussion

In this study, we have cloned and sequenced cDNAs of two novel TPM4 isoforms using RNA from human hearts (Figures 1 [Fig fig2]–3); one is the sarcomeric isoform TPM4*α* that encodes a protein with 284 amino acids and the other known as TPM4*δ* encodes a protein with 248 amino acids (Figures 1 and 4). The transcripts of these two isoforms are expressed in fetal heart, adult heart, and skeletal muscle. The expression level is somewhat higher in hearts compared to skeletal muscle (Figures 2 and 5). We were unable to detect the expression of TPM4*δ* (a ~28 kD protein) in either adult or fetal hearts by western blot analyses using CH1 monoclonal antibody that recognizes an epitope in exon 9a. However, CH1 antibody detected the expression of high molecular weight TPM proteins (~32 kDa and above) in adult and fetal hearts (Figure 6). One of the limitations in tropomyosin research is the unavailability of isoform specific antibodies. Without them, it is practically impossible to pinpoint whether the TPM4*α* protein is present in the cluster of signals at the high molecular wt region (32–40 kDa) as depicted in Figure 6. In order to gain further insight, we have performed 2D western blot analyses with CH1 antibody. The gel extracted proteins from various spots with positive signal were analyzed by LS-MS/MS. The results indicate that the spot G contains TPM4*α* protein along with TPM1*α*. It is to be noted that we have also found the expression of TPM1*κ*, TPM2*α*, and TPM3*α* in other spots (data not shown). Our future plan is to further confirm the expression of TPM4*α* protein in human striated muscles in healthy and diseased ones. We have compared the expression level of TPM4*α* transcripts in fetal and adult human hearts and are planning to compare the corresponding protein expression level in fetal and adult human hearts as well. The fact that we found more peptides specific to TPM1a than TPM4a in spot G is consistent with our bias that there is more of the former than the latter in human cardiomyocytes.

Although the expression of multiple isoforms of tropomyosin has long been known in vertebrates including humans, the precise function of each of the isoforms generated by various* TPM* genes is not well understood. For example, the expressions of TPM1*α* (the sarcomeric isoform of the* TPM1* gene) and TPM2*α* (sarcomeric isoform of the* TPM2* gene) in human hearts have been known for a long time. However, the precise role(s) of the two sarcomeric TPM proteins in cardiac muscle contraction have not been elucidated. More recent published results suggest that TPM1*α* makes up about 90% of the total sarcomeric TPM protein in human hearts. TPM2*α* and TPM1*κ* (another sarcomeric isoform of the* TPM1* gene) may constitute the remaining 10 percent of the TPM protein. Peng et al. [25] using top-down mass spectrometry confirmed that TPM1*α* constitutes 90% of the total sarcomeric tropomyosin in human hearts. They also detected a low amount of TPM2*α* and TPM1*κ* expression in human hearts. There was no mention about the expression of TPM4*α* in this paper. Marston et al. also reported the expression of TPM3*α* in human heart [26]. These authors reported that the level of expression of TPM1*α* and TPM1*κ* was 95% and 4%, respectively, of the total sarcomeric tropomyosin in human hearts. In addition, a low level of expression of TPM2*α*, TPM3*α*, and TPM1*β* (smooth muscle type tropomyosin) was also detected. Again, there was no mention about the expression of TPM4*α*.

Researchers from different laboratories using transgenic mouse models have delineated the tentative function of TPM1*α* and TPM2*α* in mammalian hearts [27–29]. In the adult healthy mouse heart the protein expression level of TPM2*α* is less than 2%. When there was 55–60% of TPM2*α* present in the myocardium of transgenic mouse heart, the time of relaxation, maximum rate of relaxation, and sensitivity to calcium changed significantly. Further altering of TPM2*α* to an even higher level in the heart of transgenic mice will cause postnatal death in between 10 and 14 days [27–29]. TPM3*α* is not expressed in mouse hearts [4]. High ectopic expression level of TPM3*α* (40–60%) can lead to hyperdynamic effect on systolic and diastolic function and decreased calcium sensitivity [30]. As TPM3*α* is not expressed in mouse heart, it is not possible to perform knockout/knockdown experiments for TPM3*α*. Again, overexpression of TPM1*κ* in a cardiac-specific manner in transgenic mouse leads to a dilated cardiomyopathy (DCM) like syndrome. Interestingly, the expression level of TPM1*κ* protein has been found to be significantly higher in hearts from DCM and heart failure patients. The functional analysis of TPM1*κ* provides a possible mechanism for the consequences of the TPM isoform switch observed in DCM and heart failure patients [31].

The functional aspect of TPM3*α* in human cardiac tissues is not known yet. Our finding of the potential expression of TPM4*α* in human heart muscles makes the situation more complicated. The most important, as well as most difficult, question yet to be addressed is whether minute expression of the sarcomeric TPM proteins such as TPM1*κ*, TPM2*α*, TPM3*α*, and TPM4*α* plays any significant role in cardiac contractility and/or cardiac function.

As rodents with truncated* TPM4* genes are not capable of producing sarcomeric TPM4*α* protein, the* in vivo* function of TPM4*α* using knockdown/knockout technology is not possible. However, one can generate transgenic mouse ectopically overexpressing human TPM4*α* protein in a cardiac specific manner and find out whether ectopic expression is altering the cardiac morphology and/or cardiac contractility. Another approach researchers have used for assigning the function of a protein is to look for its association with any human disease(s). For example, various* TPM1* and* TPM2* mutations have been implicated in hypertrophic cardiomyopathy (HCM) including familial hypertrophic cardiomyopathy (FHC) in humans [31]. Similarly, several missense mutations in* TPM2* and* TPM3* have been implicated in nemaline myopathy that suggest the functional importance of these genes or particular isoform in cellular function. To the best of our knowledge no mutation in* TPM4* gene has been implicated in any diseases of striated muscles in humans. However, one cannot rule out that this possibility was not considered before because of the belief that the* TPM4* gene in humans, as in rodents, does not produce the sarcomeric isoform TPM4*α*.

## Figures and Tables

**Table 1:** Nucleotide sequences of the primer-pairs used for conventional and qRT-PCR analyses.

Primer	Isoform	Type of PCR	Sequence
TPM4*α*-F-1 (P1+)	TPM4*α*	Both conventional & qRT-PCR	5′-CAGCCATGGAGGCCATCAAGA-3′

TPM4*α*-R-1 (P2−)	TPM4*α*	Conventional	5′-CACCATGTGAGAAGGACAGA-3′
TPM4*α*-R-2	TPM4*α*	qRT-PCR	5′-ACGAGCTGGATGCGTCGGT-3′

TPM4*δ*-F-1 (P3+)	TPM4*δ*	Conventional & qRT-PCR	5′-ATGGCCGGCCTCAACTCCCTGG-3′

TPM4d-R-1	TPM4d	Conventional	5′-CACCATGTGAGAAGGACAGA-3′
TPM4d-R-2	TPM4d	qRT-PCR	5′-ACGAGCTGGATGCGTCGGT-3′

18s rRNA-F	18s rRNA	qRT-PCR	5′-TGCTGCAGTTAAAAAGCTCGTA-3′
18s rRNA-R	18s rRNA	qRT-PCR	5-ACCAACAAAATAGAACCGCGG-3′

**Table 2:** Key to 2D gel loading and sample preparation. The sample was lyophilized and redissolved to 4 mg/mL in 1 : 1 diluted SDS boiling buffer : urea sample buffer before loading.

Gel ID #	Sample	*μ*L loaded	*μ*g loaded	Treatment
LF975 #1	HT-801 Zyagen Human Heart	150	500	Coomassie
LF975 #2	HT-801 Zyagen Human Heart	150	500	Antitropomyosin
